# The First Principal Calculation of the Temperature-Dependent Crystalline Defect Evolution in UN

**DOI:** 10.3390/ma19061163

**Published:** 2026-03-16

**Authors:** Yongheng Lu, Tingyu Sun, Zongshu Li, Yueqing Qian, Chen Chen, Lu Yu, Zheng Pan, Jing Wang, Kun Yang

**Affiliations:** 1 CNNC Key Laboratory on Fabrication Technology of Reactor Irradiation Special Fuel Assembly, Baotou 011500, China; 18047291335@163.com (T.S.); cc15326081526@163.com (C.C.);; 2 China North Nuclear Fuel Co., Ltd., Baotou 011500, China; 3 Department of Nuclear Science and Technology, Nanjing University of Aeronautics and Astronautics (NUAA), Nanjing 211106, Chinawangjing.6@nuaa.edu.cn (J.W.); 4 Key Laboratory of Advanced Nuclear Technology and Radiation Protection, Ministry of Industry and Information Technology, Nanjing 211106, China

**Keywords:** uranium nitride, first principal study, crystalline defect

## Abstract

This study systematically investigates the influence of temperature on the defect formation mechanisms in uranium nitride (UN) crystals using first-principles calculations. The formation energies and lattice distortion characteristics of various defects at 0 K and 1780 K were calculated by constructing models of perfect crystals as well as vacancy, interstitial, antisite, and divacancy defects. The results demonstrate that elevated temperatures significantly reduce defect formation energies, with interstitial and divacancy defects exhibiting negative formation energies at 1780 K, indicating a tendency for spontaneous formation. The U interstitial defect induces the most pronounced lattice expansion of 5.1% at 0 K. Furthermore, interstitial defects cause the most significant lattice distortions, while Schottky defects exhibit the lowest formation energy. The current study provides theoretical insights into the defect behavior of UN fuel under high-temperature service conditions and offers valuable guidance for optimizing sintering process parameters.

## 1. Introduction

Uranium nitride (UN) fuel is regarded as a promising candidate nuclear fuel for future space nuclear power and propulsion systems, micro-reactors, accident-tolerant fuels (ATF), and Generation IV advanced nuclear energy systems (such as lead-cooled fast reactors, LFR, and sodium-cooled fast reactors, SFR). The UN fuel enjoys advantages of high uranium density, high melting point, excellent thermal conductivity, superior high-temperature stability, and good compatibility with liquid metals [1,2,3,4]. Therefore, the properties of the UN fuel directly determine the performance of the reactor, which has been widely studied since the 1980s. The previous research on the UN fuel mainly focuses on the microstructural and phase variations induced by different temperatures and pressures during storage in air and sintering processes [4,5,6,7,8]. For instance, Olsen et al. discovered that UN undergoes a phase transition from the B1 phase to a rhombohedral structure within a pressure range of 0–34 GPa, accompanied by a 3.2% volume reduction [9]. Yang et al. (2021) systematically studied the phase degradation behavior of UN and Cr doped UN subjected to ambient air and steam at elevated temperature and discovered that the addition of Al and Cr can enhance the anti-oxidation behavior of UN [10]. With the increasing industrial application of UN, especially in the field of small modulus reactor, a fundamental understanding of the structural evolution under complex high-temperature and high-irradiation conditions is crucial for advancing its industrial application reliability. Therefore, theoretical investigations into the early-stage microstructure and defect evolution behavior of UN fuel pellets are essential for enhancing their in-service performance [11,12,13,14,15,16,17,18,19,20,21,22].

Several studies have already been carried out to understand the structure and phase transition behavior of UN subjected to temperature and pressure variations. The electronic properties, phonon dispersion relations, elastic constants, structural phase transitions, and pressure-volume equations of state of UN were studied by Modak et al. (2011) and Lopes et al. (2016) via first-principles calculations [23,24]. The phase transition properties of UN under temperature and pressure variation (0–200 GPa and 0–1500 K), revealing a structural transition from cubic to rhombohedral at 23.5 GPa, were further studied by Mei et al. (2014) by utilizing density functional theory (DFT) with the DFT + U method [13]. Moreover, the oxygen interaction of UN and O with ab initio simulations at two different crystalline planes of (001) and (110) was studied by Bocharov et al. (2013), suggesting that O tends to penetrate into the UN grain boundary with N vacancies due to negative incorporation energies and a small energy barrier [21]. In addition, the ab initio atomistic thermodynamic method can be further applied to investigate the interaction between water molecules and UN through the (110) direction under different temperatures and pressures [23]. Although several studies based on theoretical calculations, such as the work by W. Zhao et al. [25] who employed first-principles calculations based on Hubbard-corrected density functional theory (DFT + U) to systematically investigate the effects of isotropic compressive strain on defect behaviors in uranium dioxide (UO_2_) and uranium mononitride (UN), have been carried out to investigate the structural evolution of UN under temperature and pressure variation, limited studies have focused on its structural stability and thermodynamics at the microscopic scale, especially at temperatures closely related to the industrial sintering process. It is essential to reveal the underlying atomic microstructural changes, especially the defect formation process, in order to optimize the UN sintering procedure by reducing its crystalline defects.

UN spontaneously develops numerous atomic-scale defects during the high-temperature sintering process, the evolution of which significantly impacts the material’s thermodynamic properties [22,23,24]. The accumulation of crystalline defects increases atomic energy and enhances atomic mobility, leading to thermodynamic instability that induces structural changes and promotes phase transitions [13]. Therefore, investigating the defect evolution behavior of UN fuel pellets under different thermodynamic conditions is of great significance for optimizing preparation process parameters [26,27].

The work by Lan et al. [28] has systematically revealed the formation rules and fundamental characteristics of intrinsic point defects in UN crystals under antiferromagnetic ordering. It lays an important theoretical foundation for understanding the structural stability and property evolution of UN materials in nuclear reactor service environments, and also provides valuable references and guidance for the present study. This study employs ab initio first-principles calculations to explore the formation energies of different defect types in UN under varying temperature conditions (0 K and 1780 K), as well as the lattice distortion characteristics induced by these defects. Furthermore, the vibrational migration behavior of U and N atoms is analyzed based on the theoretical calculations.

## 2. Materials and Methods

### 2.1. Model Establishment

**Figure 1:**
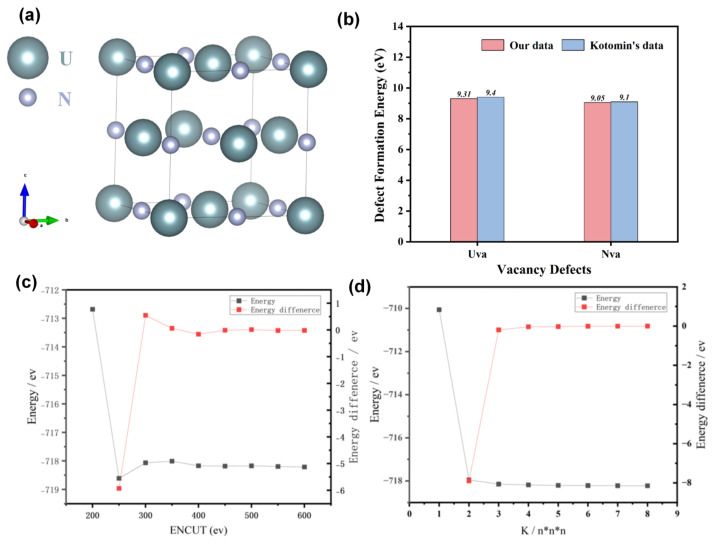
DFT model set up and validation. (**a**) the atomic model of the UN; (**b**) defect formation energy of UN as compared to the Kotomin’s data; (**c**) Convergence test of plane-wave cutoff energy (ENCUT); (**d**) Convergence test of K-point mesh density. Note: K/n*n*n represents the K point density used for the structural optimization and calculation.

The UN at room temperature maintains a cubic B1 structure (space group Fm-3m, No. 225), where U and N atoms occupy the vertex and face-centered positions of the face-centered cubic lattice, respectively, with a typical lattice constant of a = 0.489 nm [11]. In the current study, a 2 × 2 × 2 supercell model contains a total of 32 U atoms (coordinates: (0, 0, 0), (0, 0, 0.5), etc.) and 32 N atoms (coordinates: (0.5, 0.5, 0.5), (0.5, 0, 0), etc.), with a total atoms of 64 and a supercell size of 0.978 nm × 0.978 nm × 0.978 nm was constructed in order to balance computational accuracy and modeling efficiency (Figure 1a). The structural optimization of the perfect supercell was performed before the calculation and further compared with the past literature in order to validate the mathematical model (Figure 1b–d). Specifically, the exchange-correlation functional employed the Perdew-Burke-Ernzerhof (PBE) generalized gradient approximation (GGA). The DFT + U method was applied with a U value of 4.0 eV (determined by comparing the cohesive energy and lattice constant of UN under different U values (3.5–4.5 eV), where 4.0 eV yielded the best agreement with experimental data) [16]. The as-calculated defect formation energy was approximately the same as compared to Kotomin’s data, suggesting the accuracy of the current model (Figure 1b). The interaction between ion cores and valence electrons was described using the valence electron configurations of U and N atoms set as 6s^2^6p^6^5f^2^6d^2^7s^2^ and 2s^2^2p^3^, respectively. Systematic convergence tests on the cut-off energy and k-point grid demonstrate that a cut-off energy of 500 eV and a 5 × 5 × 5 Monkhorst-Pack k-point mesh can limit the total energy difference in the system to within 0.005 eV, which meets the computational accuracy requirements of the present study. The energy convergence criterion was set to 10^−5^ eV/atom with the atomic force convergence threshold to 0.01 eV/Å. Results suggested the optimized lattice constant of UN was 0.491 nm, while the experimental value of the lattice constant is 0.489 nm, showing a relative error of 0.41% [13]. The calculated cohesive energy of −12.90 eV/atom, which is in good agreement with the result (−12.85 eV/atom) reported by Kotomin et al. (2017), confirms the reliability of the perfect UN crystal model [6]. Ab initio molecular dynamics simulations were further carried out in the NVT canonical ensemble using a Nose–Hoover thermostat to control the system temperature. The energies of both defect-free and defective crystals were calculated. The time step was set to 0.5 fs, and the simulation was performed for 10,000 steps, corresponding to a total simulation time of 5 ps. The energies during the last 2000 steps were extracted and averaged to obtain statistically averaged properties from the ab initio molecular dynamics simulation.

Five typical UN defect models were constructed and studied based on the optimized perfect supercell in order to systematically understand the influence of different crystal defects on the structural properties of UN, such as vacancy defects, interstitial defects, antisite defects, and diatomic defects (Frenkel and Schottky defects). The defect sites were selected at crystallographically high-symmetry positions to ensure the representativeness of the calculation results. After constructing all defect models, the NVT ensemble (supercell volume constant) was used during the calculation, followed by structural relaxation, with only atomic positions optimized during relaxation to simulate the volume constraint conditions of fuel pellets in actual service environments.

### 2.2. Calculation of Defect Formation Energy

The defect formation energy (E_f) is a key parameter used to represent the defect stability, defined as the minimum energy required to form a defect. The calculation formula is given by [17,18,19,20]:

$$E_{trap}=E_{def}-E_{perf}-\sum_{I}n_{i}^{X}E_{i}$$

where $E_{trap}$ is the formation energy of the studied defect; $E_{def}$ is the ground-state total energy of the supercell containing the defect; $E_{perf}$ denotes the total energy of the supercell of perfect UN (containing 64 atoms in this study); $n_{i}^{X}$ is the number of i-type atoms added (n_i_ > 0) or removed (n_i_ < 0) to form the defect; and $E_{i}$ is the total energy of isolated i-type atoms such as uranium (U) and nitrogen (N).

### 2.3. Electron Structure

The electronic structure analysis was performed on the optimized defect models aimed to elucidate the microscopic mechanisms of defect formation and evolution. Density of States (DOS): The total density of states (TDOS) and partial density of states (PDOS) were calculated to analyze the influence of defects on electronic state distribution [12]. Charge Density Distribution: Isosurface plots were generated to visualize charge transfer and bonding characteristics between atoms. Electron Localization Function (ELF): The ELF value ranges from 0 to 1, where ELF ≈ 1 indicates highly localized electrons (e.g., covalent bonds or lone pairs), and ELF ≈ 0.5 represents free-electron-gas behavior (e.g., metallic bonds), which can be used to characterize the bonding type and electron localization of U-N bonds. All calculations were performed using the CASTEP v20.1 (Cambridge Serial Total Energy Package) software [12].

## 3. Results

### 3.1. Crystal Defect Behavior of Uranium Nitride at 0 K (Absolute Zero) Reference Temperature

Defect properties are primarily determined by electronic structure and lattice geometry at absolute zero (0 K) with negligible atomic thermal vibrations. Therefore, the defect formation energies, lattice distortions, and electronic structure features were further analyzed.

**Figure 2:**
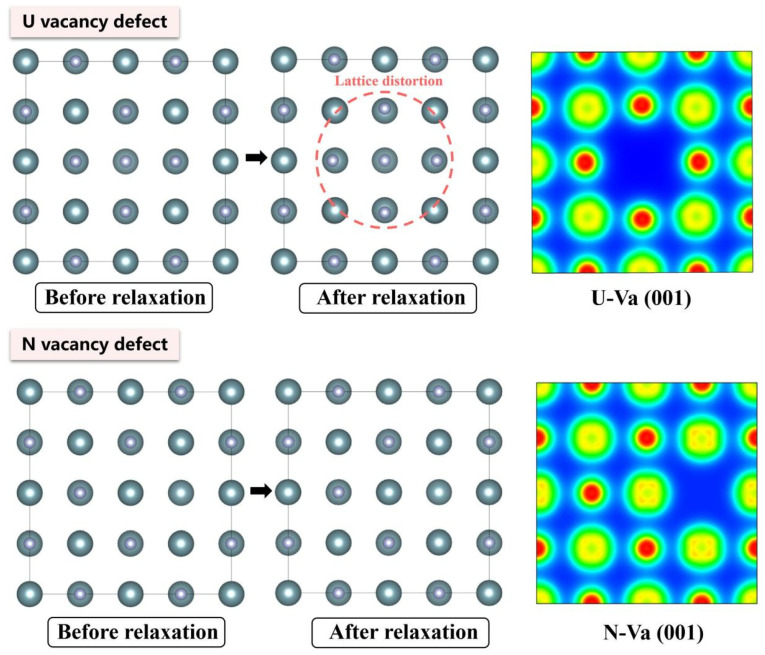
Evolution of the UN vacancy defect at 0 K.

**Figure 3:**
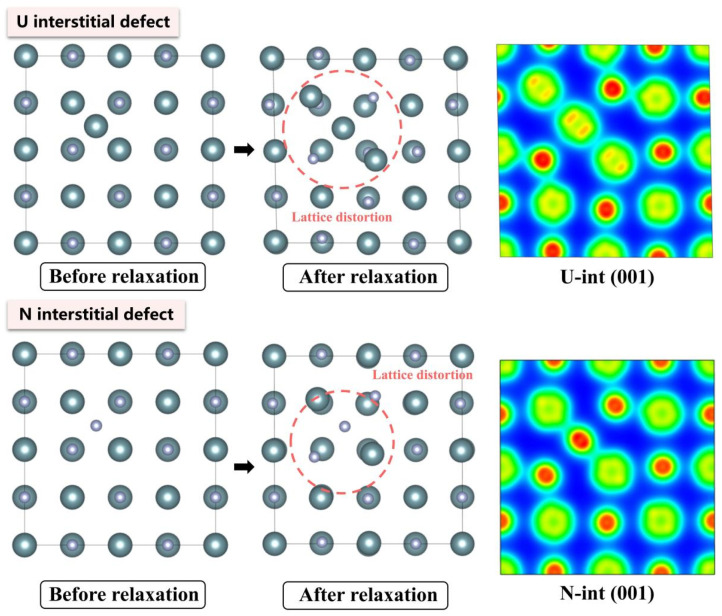
Evolution of UN interstitial defect at 0 K.

**Figure 4:**
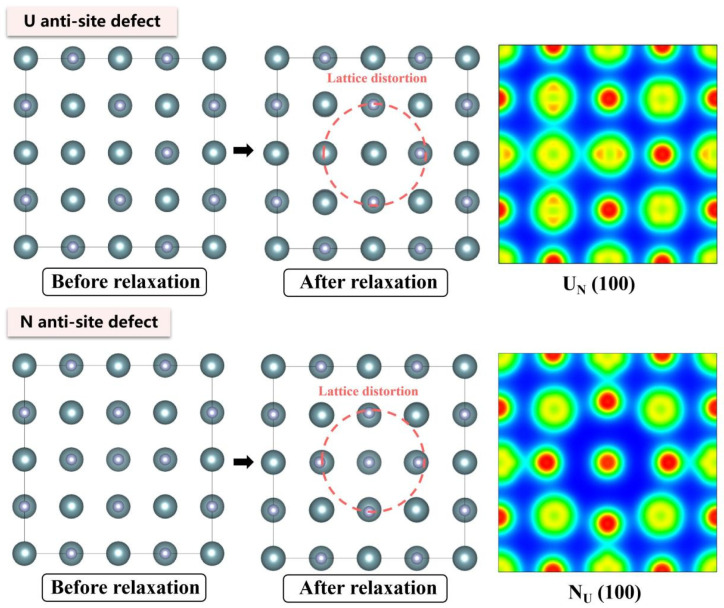
Evolution of UN antisite defect at 0 K.

**Figure 5:**
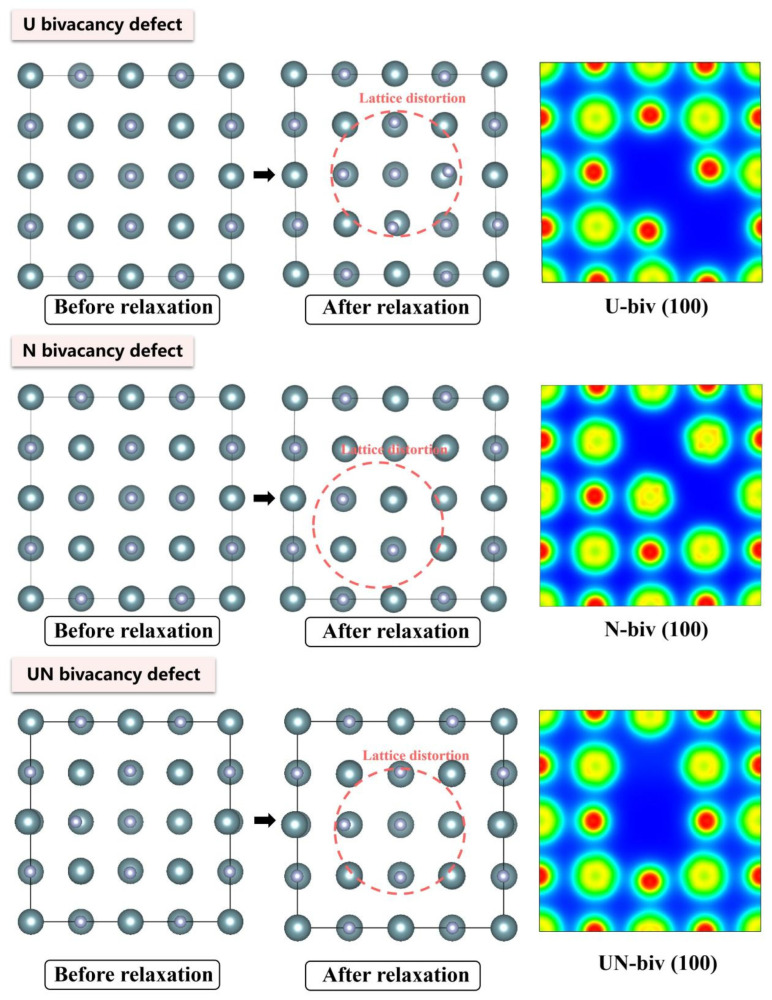
Evolution of UN bivacancy defect at 0 K.

**Figure 6:**
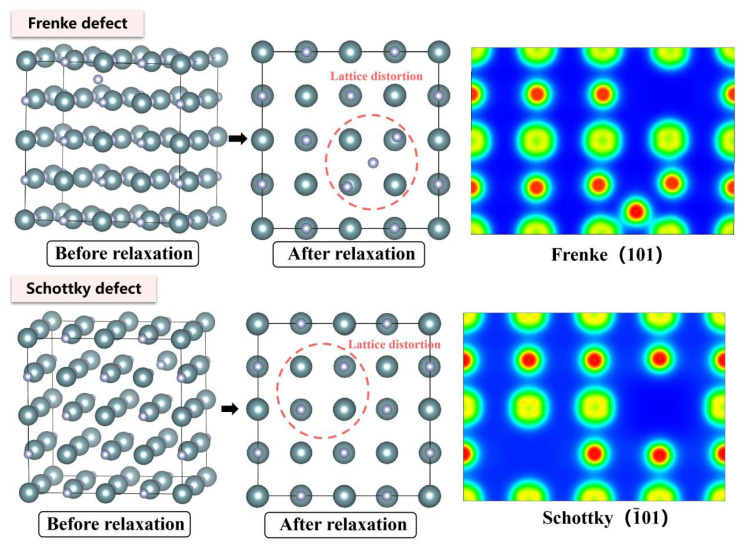
Evolution of UN Frenkel and Schottky defect at 0 K.

**Figure 7:**
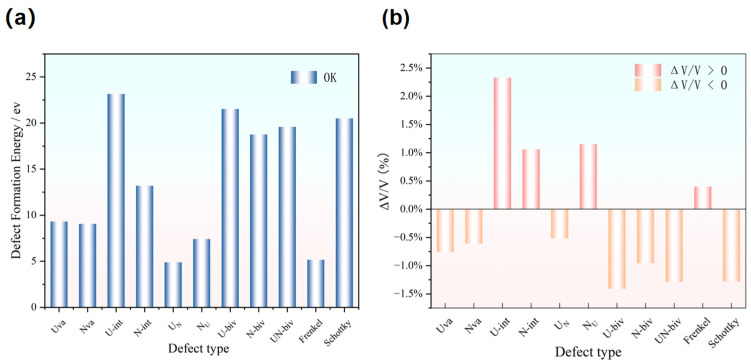
Defect formation energy of different types of defect at 0 K (**a**) and lattice volume expansion ratio (**b**).

The electronic density distributions and formation energy analyses for various defects in UN at 0 K are presented in Figure 2, Figure 3, Figure 4, Figure 5 and Figure 6, while the calculated defect formation energies and unit cell expansion rates are shown in Figure 7. The results indicate that the electron density on U atoms exhibits an isotropic distribution. On the (100) crystal plane, the electronic polarization of each U atom primarily orients toward its four nearest-neighbor U atoms with opposite spin directions, suggesting that UN crystals contain not only U-N bonds but also significant U-U interactions. Bader charge analysis reveals that the negative charge (absolute value) of N atoms adjacent to a U vacancy decreases from approximately 1.52 e in perfect UN crystals to about 1.48 e for the U single vacancy defects, while the positive charge of U atoms nearest to a N vacancy decreases to an average of approximately 1.37 e, respectively. On the contrary, the corresponding formation energy of N vacancy (Nva, 9.31 eV) exceeds that of U vacancy (Uva, 9.05 eV) by 0.26 eV, indicating that N atoms need to overcome a higher energy barrier to leave their original lattice sites, implying that it is more difficult to form vacancies. The calculated defect formation energy aligns well with previous research by Kotomin et al. (2017), suggesting strong hybridization between N 2p and U 5f orbitals (Figure 7a), which can be attributed to the significant covalent character of the U-N bond [6]. Meanwhile, the defect formation energy of divacancy all exceeds twice that of single vacancies, specifically, adjacent U-divacancy (U-biv) formation energy is 19.58 eV (2.16 × Uva), while adjacent N-divacancy (N-biv) is 18.76 eV (2.02 × Nva). On the other hand, separated divacancies exhibit slightly higher formation energies (U-biv:20.32 eV; N-biv:19.45 eV) than adjacent configurations, suggesting weak attractive interactions between neighboring defects that reduce formation energy, which can be attributed to the overlapping lattice distortion regions between adjacent vacancies partially relieving lattice strain, thereby stabilizing the defect system [16].

Both the U-int and N-int interstitial defects cause significant displacement of surrounding atoms in UN, with the nearest-neighbor U atoms being displaced outward by 0.343 Å from the U-int position, while the nearest-neighbor N atoms are displaced outward by 0.273 Å from the N-int position, which can be reflected from the atomic structure before and after relaxation as denoted in Figure 2, Figure 3, Figure 4, Figure 5 and Figure 6. For the U-int defect, the interstitial U atom carries approximately 0.25 e, less than half the charge of a normal U atom (1.52 e), and the electron density of the U-int atom shows significant overlap with adjacent U atoms. For the N-int defect, the interstitial N atom gains 1.16 e from surrounding U atoms, which is less than the 1.52 e of a normal N atom. The corresponding defect formation energy of U-int (23.15 eV) is much higher than that of N-int (13.19 eV), with a difference of 9.96 eV, which can be attributed to the higher atomic radius of U (0.138 nm) than that of N (0.075 nm). The higher atomic radius of U causes severe lattice compression when inserted into octahedral interstitial sites (radius 0.065 nm), which requires higher energy to overcome steric hindrance. Furthermore, the antisite formation energy of N (13.19 eV) is higher than that of the U antisite (10.90 eV). Specifically, when N atoms occupy U lattice sites, a smaller atomic radius is created, leading to a mismatch with the coordination environment of surrounding U atoms, further resulting in an increase in lattice distortion and higher formation energy. Bader charge analysis that the antisite atom in U_N defects does not lose electrons but instead gains approximately 0.40 e from neighboring U atoms. The clustering of U atoms around the U_N antisite defect enhances the uranium charge density in this region. For N_U antisite defects, the bonding interaction between nearest-neighbor N atoms and next-nearest U atoms strengthens significantly. In this configuration, the N_U antisite atom acquires a substantial negative charge (~0.82 e), while the nearest-neighbor N atoms surrounding the defect exhibit pronounced outward displacement from the central defect site. Divacancies induce local lattice distortions and charge redistribution, affecting bulk properties to varying degrees since divacancies serve as primary initiators of subsequent high-temperature phase transitions in UN [18]. Specifically, U–U divacancies cause surrounding N atoms to bond more tightly with adjacent U atoms, forming an extended dumbbell-shaped void.

The N interstitial atom gains ~0.24 e from neighboring U atoms in Frenkel defects, accompanied by significantly enhanced bonding interactions with surrounding U atoms. For Schottky defects, U vacancies cause electron density to accumulate along diagonal directions among adjacent U atoms, while N vacancies lead to electron density clustering around the vacancy sites toward the N-atom directions. The Schottky defect exhibits the lowest formation energy (4.88 eV), substantially lower than Frenkel defects (U-Frenkel: 7.20 eV; N-Frenkel: 6.85 eV), which can be explained by the “cooperative vacancy formation” mechanism. Specifically, simultaneous removal of U and N atoms allows their respective lattice distortion fields to compensate, thereby reducing the overall energy barrier mutually. Conversely, Frenkel defects exhibit additive lattice distortions from both interstitial atoms and vacancies, resulting in higher formation energies.

### 3.2. Lattice Distortion

The volume expansion rate, which was characterized by the maximum displacement of atoms surrounding the defect, can be used to evaluate the degree of lattice distortion. (Figure 7b). Among these, U-int exhibits a relative volume expansion of 5.10% with a maximum atomic displacement of 0.32 Å, which can be attributed to the strong compressive force on its four nearest-neighbor N atoms derived from the interstitial U atom and further leads to the outward displacement from the interstitial site. For comparison, the divacancy defects cause greater distortion than single vacancies, with adjacent divacancies demonstrating higher distortion rates than separated configurations, suggesting that defect interactions amplify lattice distortion [17]. The distortion rate of Frenkel defects (2.53%) is notably higher than that of Schottky defects (1.00%). The discrepancy can be attributed to the “vacancy-interstitial” pair in Frenkel defects, which concentrates lattice stress, whereas the uniformly distributed vacancy pairs in Schottky defects facilitate more effective stress relaxation. Since lattice distortion predominantly occurs within 1–2 lattice constants around the defect core, with atomic displacements beyond this region measuring less than 0.01 Å, suggesting defect-induced distortion effects are featured with short-range.

### 3.3. Crystal Defect Behavior of Uranium Nitride (UN) at 1780 K, High Temperature

**Figure 8:**
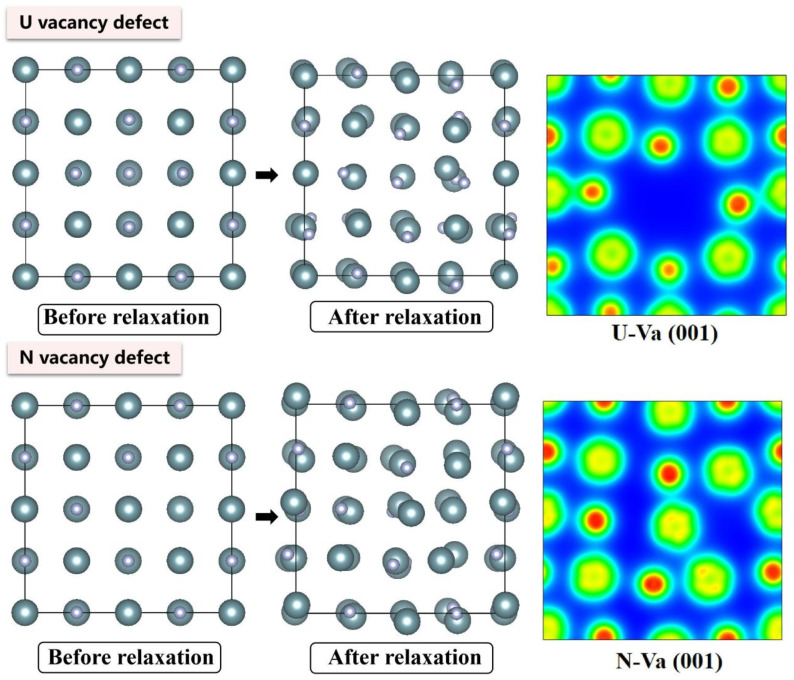
Evolution of UN vacancy defect at 1780 K.

**Figure 9:**
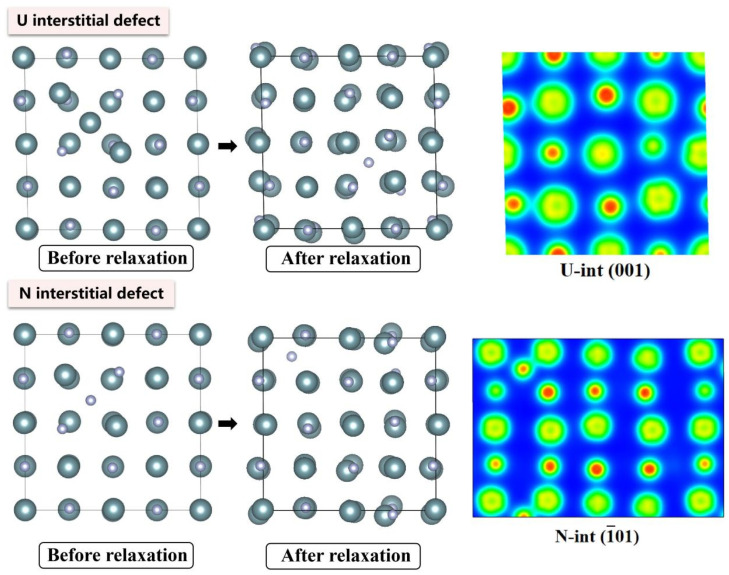
Evolution of the UN interstitial defect at 1780 K.

For comparison, the defect formation energy and localized electron property were further calculated at 1780 K in order to simulate the typical sintering temperature range for UN pellets. The average positive charge of nearest-neighbor U atoms reduces to ~1.16 e as compared to 0 K in N-vacancy structures, suggesting significant temperature-dependent modifications in defect charge distribution [22,23]. Notably, the defect formation energy was temperature dependent with U vacancy (Uva) formation energy remains at 4.9 eV, N vacancy (Nva) formation energy decreases remarkably to nearly 0 eV by increasing temperature to 1780 K (Figure 8). Compared to pre-relaxation states, significant displacements of neighboring atoms can be observed for the interstitial defects (U-int and N-int) at 1780 K (Figure 9). The formation of a U vacancy attracts surrounding N atoms toward the vacancy site and simultaneously results in a N interstitial atom. The lattice then underwent substantial displacement of approximately 7 Å along the z-direction. Bader charge analysis reveals that this interstitial N atom carries a higher charge of 1.67 e compared to other N atoms. For U interstitial defects, neighboring N atoms exhibit slightly elevated charges averaging 1.52 e. At elevated temperature, the N interstitial atom diffuses outward and stabilizes at a position far from the unit cell center, with a prominent 2.6 Å displacement along the y-direction as compared to the 0 K result. This N interstitial carries a reduced charge of 1.12 e as compared to the scenario at 0 K, confirming a temperature-induced weakening tendency in U-N covalent bonding. In addition, interstitial formation energies of U and N decreased to negative values (−1.4 eV for U-int and −6.2 eV for N-int) with a significant reduction compared to the 0 K value, suggesting spontaneous defect formation occurred at elevated temperature.

**Figure 10:**
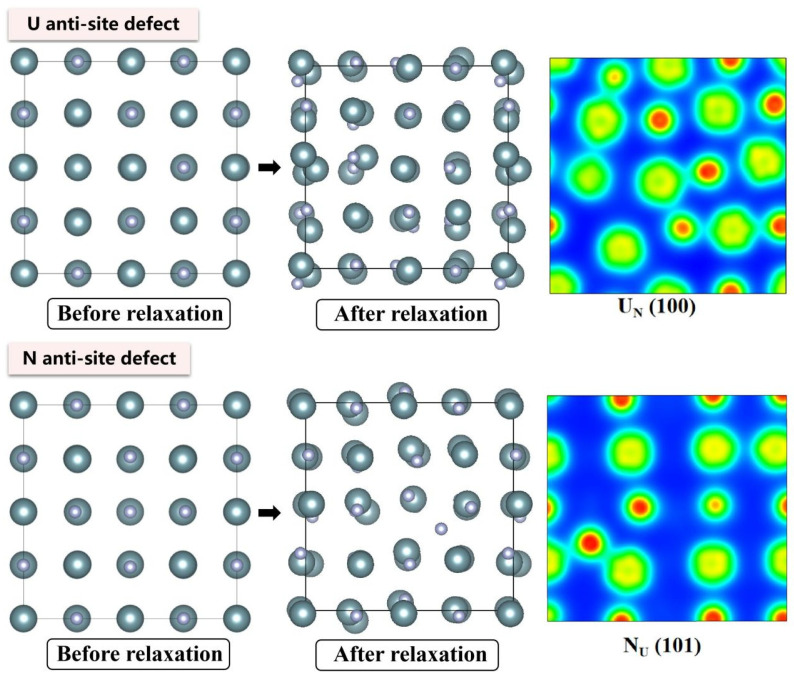
Evolution of UN antisite defect at 1780 K.

Compared to 0 K conditions, atoms neighboring UN and NU antisite defects at elevated temperatures exhibit pronounced displacement away from the central defect site while engaging in strong charge interactions with adjacent atoms (Figure 10). Bader charge analysis indicates that the antisite U atom in U_N defects gains approximately 0.35 e from surrounding U atoms, demonstrating the enhanced local U charge density due to the U clustering around the antisite. Electron Localization Function (ELF) analysis further reveals that U antisite formation induces displacement of other unit cell atoms by increasing their bonding tendency. For N_U antisite defects, the antisite N atom progressively migrates from its initial position and further forms an interstitial site near the original location with reduced charge (~1.1 e, approximately 0.4 e lower than surrounding N atoms) according to the AIMD simulation, which can be attributed to the diminished U coordination. On the other hand, the corresponding defect formation energies decrease to 1.2 eV for U_N antisites and 6.9 eV for N_U antisites, lower than the scenario at 0 K.

**Figure 11:**
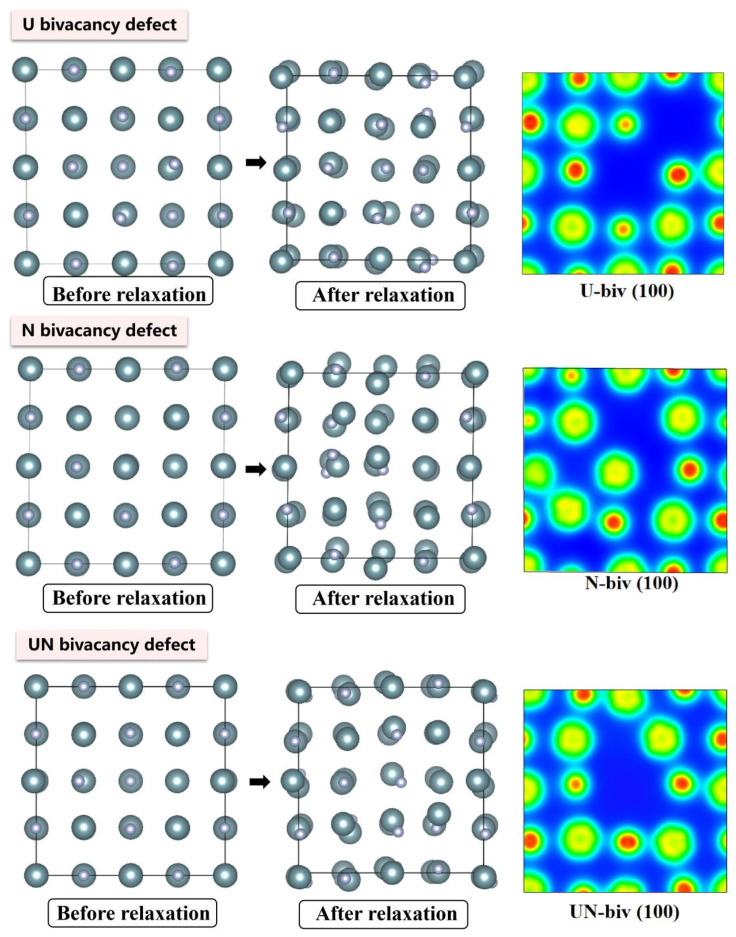
Evolution of UN bivacancy defect at 1780 K.

The divacancy defects were further calculated at 1780 K and compared to the previous results at 0 K (Figure 11). Results demonstrate that the neighboring N atom charge was reduced to 1.46 e with the formation of a U vacancy at 1780 K, as compared to 0 K, with charge overlap occurring between the nearest N atom to the U vacancy and adjacent U atoms according to the analysis of Bader charge. Moreover, the U atoms near N vacancies exhibit decreased average charge (1.13 e vs. 1.51 e in perfect crystals) alongside enhanced bonding tendencies near the U atom. N atoms gradually moved to U vacancy sites, with U atoms simultaneously aggregating around N vacancies for UN divacancies. The Bader analysis further indicates reduced charge transfer in U atoms near N vacancies (average: 1.21 e), a 0.05 e charge gain in N atoms adjacent to N vacancies, and decreased charge in N atoms surrounding U vacancies (average: 1.44 e). As a result, the divacancy formation energies decreased dramatically to U-biv (6.4 eV) and N-biv (11.1 eV), representing a 40% reduction versus 0 K values and confirming substantial thermal influence on defect stability.

**Figure 12:**
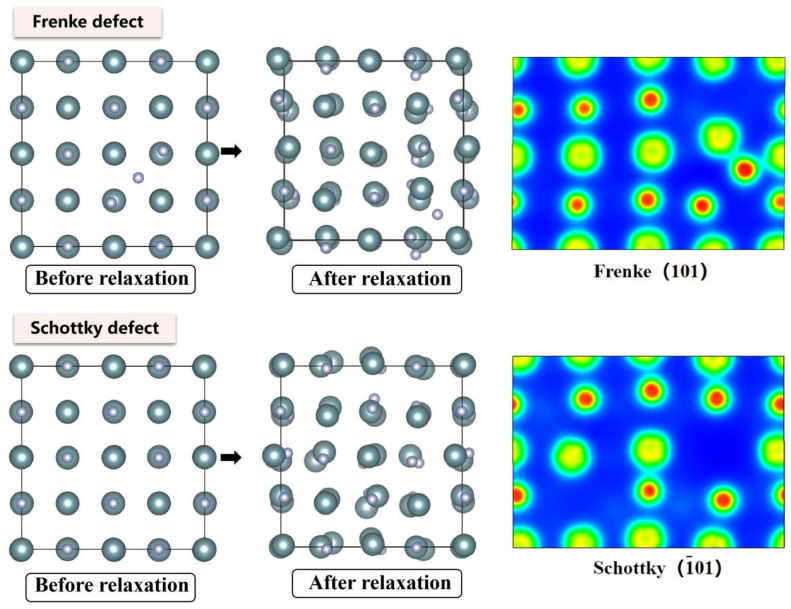
Evolution of UN Frenkel and Schottky defect at 1780 K.

**Figure 13:**
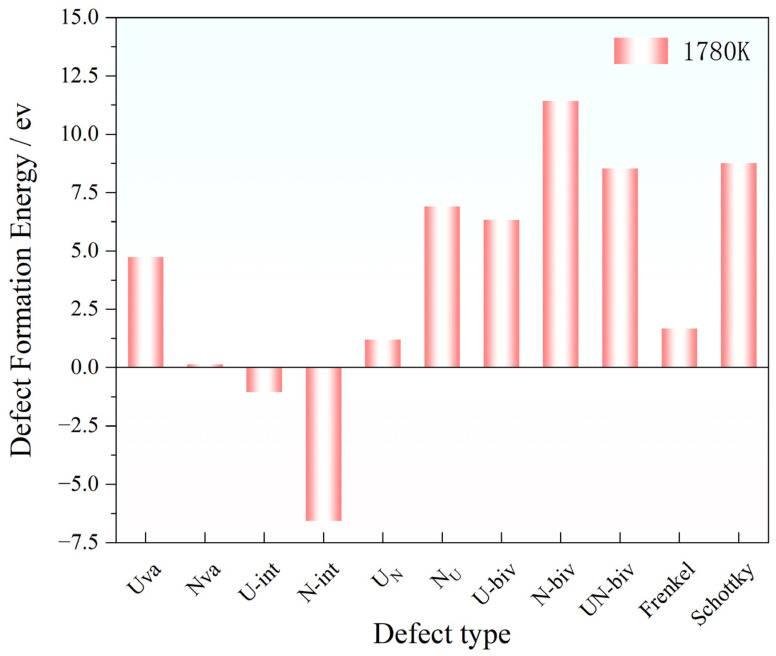
The defect formation energy for different types of defects at 1780 K.

The N interstitial atom relocates to generate a defect site in the lower-right region relative to its initial position in the Frenkel defect scenario (Figure 12). The adjacent U atoms agglomerated together with the formation of an N vacancy, with the enhancement in the bonding tendency between the U atoms and the interstitial N atom. For comparison, a similar agglomeration of atoms toward the vacancy can be observed for the Schottky defects. Bader charge analysis reveals that U vacancies prompt charge accumulation along diagonal directions among neighboring U atoms, alongside the displacement of N atoms toward adjacent U positions. The formation of U vacancy results in the reduction in the N atomic charge (1.46 e), whereas N vacancies trigger collective clustering of U atoms accompanied by diminished charge transfer (average U charge: 1.29 e), ultimately resulting in the 100% reduction in Frenkel defect formation energy and Schottky defect formation energy (1.8 eV and 8.1 eV at 1780 K, separately) (Figure 13).

## 4. Discussion

**Figure 14:**
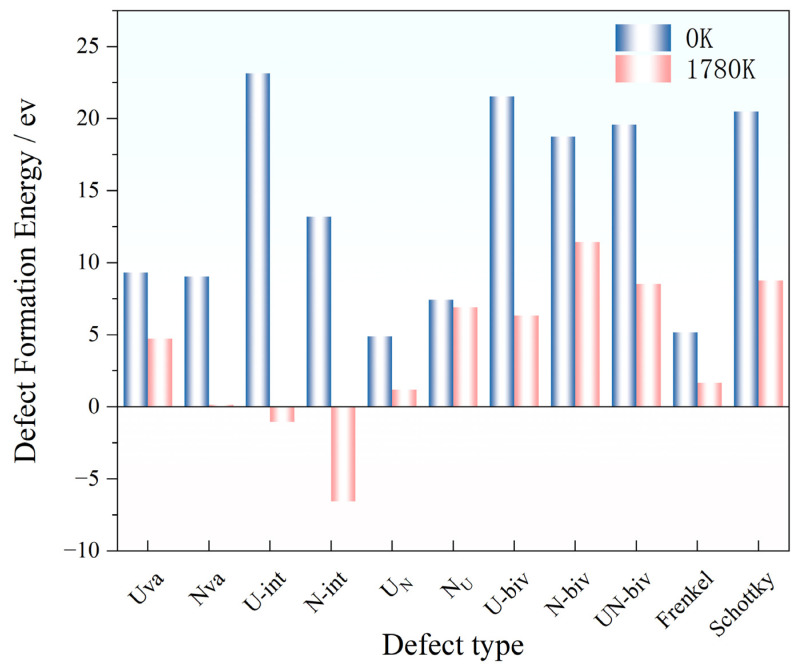
Defect formation energies of different defects in UN at 0 K and 1780 K.

As can be seen from Figure 14, the variety of defect formation energies decreases significantly by increasing temperature, indicating the temperature-driven decreases in the energy threshold for forming different crystalline defects and consequently facilitates defect generation [11]. Notably, the interstitial defect formation energies for both U and N become negative at 1780 K, suggesting spontaneous formation of interstitial defects, further confirming that the interstitial defect is the most readily formed defect type, which profoundly impacts the unit cell volume of UN upon generation. Meanwhile, Frenkel and Schottky defects within divacancy systems are the most temperature-sensitive type of defect, with 100% reduction in the formation energy observed at elevated temperatures.

The impact of temperature on the defect formation mechanisms of UN can be explained by two mechanisms: “energy supply” and “lattice environment” [6]. Specifically, elevated temperatures provide additional thermal activation energy that reduces defect formation barriers, exemplified by U vacancy (Uva) formation energy decreasing from 9.05 eV at 0 K to 7.98 eV at 1780 K, enabling atoms to overcome lattice constraints. Moreover, significant lattice expansion (2.24% at 1780 K) can be induced by increasing the temperature. In addition, the enlargement of atomic distance and interstitial volume can be observed at elevated temperature with simultaneous decreases in steric hindrance for interstitial/antisite defects [12]. Specifically, the U-int defect formation energy decreases by 12.2% (0 K→1780 K) while more than 100% reduction in the formation energy can be observed for Frenkel/Schottky defects. In addition, the interatomic interactions were weakened with the intensified lattice vibrations, diminishing U-N bond covalency (ELF decline from 0.75 to 0.62) and further leading to the reduction in electronic structure barriers for defect formation [13,26].

However, the different types of defects exhibited different responsiveness to the temperature. Specifically, divacancy defects (particularly Frenkel defects) exhibit the greatest formation energy reduction (16.7%), while single vacancies show the smallest decrease (8.4%). The distinct discrepancy can be attributed to the atomic migration and rearrangement during the divacancy formation, which is highly sensitive to temperature. On the other hand, single vacancies entail only atomic removal with limited thermal dependence. Elevated temperatures significantly lower defect formation energies, yielding the lowest Schottky defect energy (4.88 eV) at 1780 K, while Frenkel defects induce the most pronounced lattice distortion (2.53% expansion rate). Electronic structure analysis confirms U-N bond covalency governs the difference in the 0 K defect energetics, with higher N vacancy energy attributed to strong N 2p–U 5f hybridization [25]. The temperature drives the decrease in covalency (ELF 0.75→0.62), while simultaneously increasing ionicity, thus reducing the electronic barriers to defect formation [19]. Overall, the crystalline defect evolution critically impacts UN service performance: lattice distortion degrades mechanical strength, defect scattering deteriorates thermal conductivity, and high-temperature defect aggregation may exacerbate irradiation damage. Thus, engineering protocols must precisely control UN fuel pellet sintering temperatures to mitigate defect formation and clustering [27].

## 5. Conclusions

This study fundamentally investigated the defect evolution of different types of defects in UN crystal at elevated temperature via first-principles calculations. Specifically, the defect formation energies and lattice impacts for vacancy, point, and antisite defects relevant to sintering processes were revealed:

(1) At the 0 K absolute temperature, statistical analysis reveals that interstitial defects induce the most severe unit cell volume expansion in UN, followed by antisite defects, while divacancies and Schottky defects exert minimal influence on lattice expansion/contraction.
(2) Density functional theory + U calculations at 0 K characterize six monovacancy and five divacancy defects. Higher formation energies indicate greater difficulty in defect generation. Among stoichiometric UN defects, U interstitial (Uint) emerges as the most favorable monovacancy (23.15 eV), though its formation drastically distorts the unit cell volume. U-biv divacancy exhibits the lowest divacancy formation energy (21.53 eV). Frenkel pairs and antisite defects form most readily yet contribute minimally to overall lattice distortion;
(3) At 1780 K, formation energies decrease significantly across all defect types, with interstitials and divacancies showing the most pronounced reductions. This confirms enhanced defect generation at high temperatures where thermal atomic motion provides the driving force.

## Data Availability

The original contributions presented in this study are included in the article. Further inquiries can be directed to the corresponding authors.
